# Exposure to the antimicrobial peptide LL-37 produces dendritic cells optimized for immunotherapy

**DOI:** 10.1080/2162402X.2019.1608106

**Published:** 2019-05-01

**Authors:** Emily Gwyer Findlay, Andrew J. Currie, Ailiang Zhang, Jana Ovciarikova, Lisa Young, Holly Stevens, Brian J. McHugh, Marta Canel, Mohini Gray, Simon W.F. Milling, John D.M. Campbell, John Savill, Alan Serrels, Donald J. Davidson

**Affiliations:** a University of Edinburgh Centre for Inflammation Research, Queen’s Medical Research Institute, Edinburgh, UK; b School of Veterinary and Life Sciences, Murdoch University, Perth, Western Australia, Australia; c Institute of Infection, Immunity and Inflammation, University of Glasgow, Glasgow, UK; d Scottish National Blood Transfusion Service, Heriot Watt Research Park, Edinburgh, UK

**Keywords:** Immunotherapy, dendritic cells, cathelicidin, CD103, PD1, cancer, host defense peptide, CLEC9A, CD86, CD141, cross-presentation

## Abstract

Immunization of patients with autologous, *ex vivo* matured dendritic cell (DC) preparations, in order to prime antitumor T-cell responses, is the focus of intense research. Despite progress and approval of clinical approaches, significant enhancement of these personalized immunotherapies is urgently needed to improve efficacy. We show that immunotherapeutic murine and human DC, generated in the presence of the antimicrobial host defense peptide LL-37, have dramatically enhanced expansion and differentiation of cells with key features of the critical CD103^+^/CD141^+^ DC subsets, including enhanced cross-presentation and co-stimulatory capacity, and upregulation of CCR7 with improved migratory capacity. These LL-37-DC enhanced proliferation, activation and cytokine production by CD8^+^ (but not CD4^+^) T cells *in vitro* and *in vivo*. Critically, tumor antigen-presenting LL-37-DC increased migration of primed, activated CD8^+^ T cells into established squamous cell carcinomas in mice, and resulted in tumor regression. This advance therefore has the potential to dramatically enhance DC immunotherapy protocols.

## Introduction

Dendritic cells (DCs) play a critical role in activating cytotoxic CD8^+^ T-cell responses against tumors (reviewed in^1^) and as a consequence there is significant interest in developing DC-based vaccines as personalized cancer therapies.^1–3^ This strategy has led to the approval of the immunostimulant Sipuleucel-T, a mixed preparation of myeloid cells treated to induce DC differentiation, for the treatment of hormone refractory metastatic prostate cancer.^4^ In general, clinical trials have proven DC-based therapies to be well tolerated, enabling patients to maintain quality of life;^4,5^ however, overall their clinical efficacy has been disappointing. A number of factors are thought to contribute to this lack of efficacy, including (1) an inability of the injected DC to migrate effectively to the lymph nodes following adoptive cell transfer and (2) an inability to sufficiently stimulate robust T-cell activation in a tumour setting. Therefore, an improved understanding of how to optimize the *ex vivo* generation of DCs with potent antitumor activity, enabling rapid, cost-effective expansion from simple preparations of a patient’s blood cells, is needed if this therapeutic strategy is to impact on the treatment of patients with cancer.

CD103^+^ DCs (CD141^+^ in humans^6^) have emerged as a fundamentally important subset that excels in cross-presentation, CD8^+^ T-cell activation and the induction of antitumor immunity.^7–14^ Expression of co-stimulatory molecules including CD86 and CD40 enables these DC to strongly activate cognate T cells, while expression of CLEC9A and XCR1 facilitates collection of antigen from dead cells and its cross-presentation to CD8^+^ T-cells.^8,15–17^ Expression of CCR7 is required for migration of DC bearing tumor antigen to lymph nodes^18^ and production of IL-12 for development of efficacious CD8^+^ T-cell immunity in patients with cancer.^19^ Indeed, it is now established that these CD103^+^/CD141^+^ DC are the main antigen presenting cell subset responsible for migrating to lymph nodes and activating antitumor CD8^+^ T-cell responses.^18,20–22^ Therefore, CD141^+^ DCs have become an attractive candidate for development as a cell-based cancer therapy. However, this cell population is rare in peripheral blood, typically 0.03–0.08% of all circulating peripheral blood mononuclear cells (PBMCs). Hence, approaches that enable the rapid expansion of cells with the key properties of CD141^+^ DC are required if they are to be developed as a viable cancer therapy.

Here, we identify a novel role for the antimicrobial host defense peptide LL-37 in directing the expansion and differentiation of DCs in culture toward an “enhanced” CD141^+^/CD103^+^-like phenotype with dramatically improved antitumor activity. We show that human and murine LL-37-DCs exhibit increased migratory capacity toward XCR1 and CCR7 ligands, and enhanced co-stimulatory and cross-priming/presentation properties, resulting in robust antitumor CD8^+^ PD1^+^ T-cell responses and even tumor regression. Therefore, LL-37-mediated reprogramming of DCs drives differentiation and expansion of an *ex vivo* generated population with enhanced functionality that may be therapeutically beneficial.

## Results

### LL-37 increases the *ex vivo* generation of CD103^+^DC in a BATF3-dependent manner

**Figure 1.**
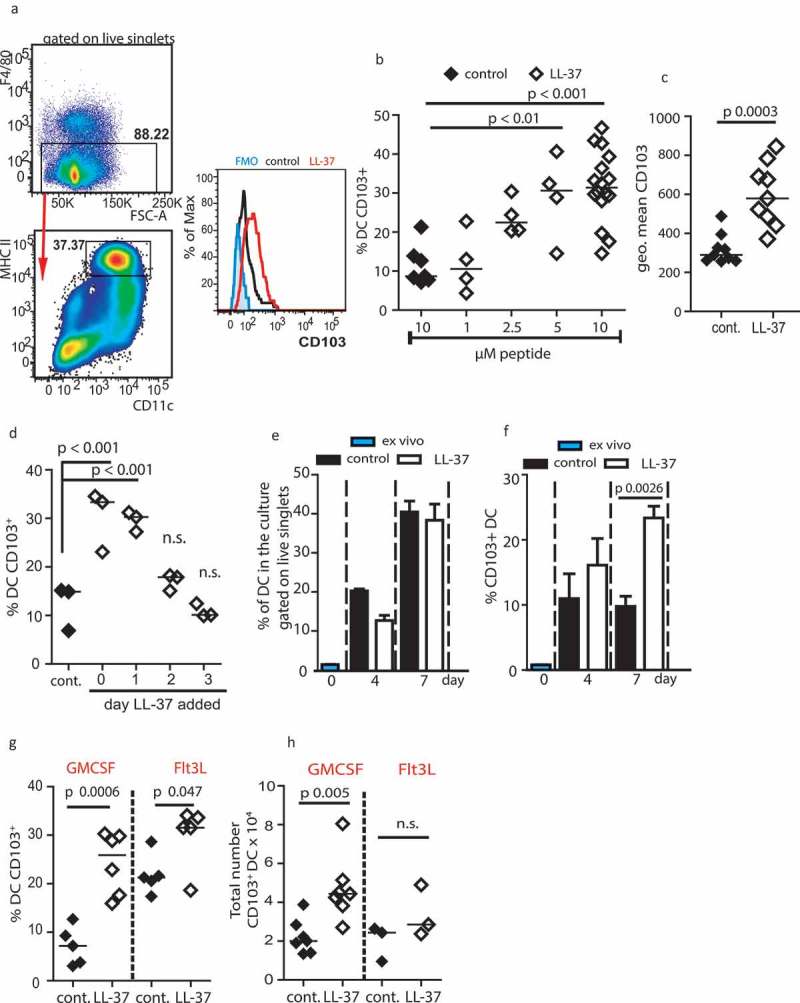
LL-37 promotes generation of CD103^+^ DC. Bone marrow from adult C57Bl6/J mice was cultured for 7 days with 20 ng/mL GM-CSF (a–h) or 20 ng/mL Flt3-L (g–h) before CD103 expression on DC generated was assessed by flow cytometry. (a–c) DC were exposed to 10 µM LL-37 or scrambled control peptide; (d) LL-37 was added on days shown; (e, f) the proportion of DC and of CD103^+^ DC in cultures was assessed over time; (g–h) growth factors GM-CSF and Flt3-L were compared. Data shown are mean ± standard error or individual data points with line at median. Statistical tests used: (b) one-way ANOVA with Dunnett’s post-test comparing all to control, *n* = 4 −16 mice; (c) two-tailed *t*-test, *n* = 9; (d) one-way ANOVA with Dunnett’s post-test comparing all to control, *n* = 3; (e, f) paired two-tailed *t*-test, *n* = 3–5; (g, h) two-tailed *t*-test, *n* = 3–6.

To assess the capacity of the antimicrobial host defense peptide LL-37 to modulate DC differentiation and function in a model system, bone marrow from wildtype (WT) C57Bl/6JOlaHsd mice was cultured for 7 days in the presence of 20 ng/mL recombinant GM-CSF. DCs were identified as shown in Figure 1(a) (as per published methodology^23^). DC cultured in the presence of 10 µM LL-37 (LL-37-DC) had a strikingly higher proportion of CD103^+^ cells (Figure 1(a, b)), compared to those cultured with control scrambled LL-37, and a higher total number of CD103^+^ DC (mean 22.5 ± 8.9 x 10^3^ control DC; 47.0 ± 16.5x 10^3^ LL-37-DC per well, *p* = 0.007 by paired *t*-test). The scrambled control peptide had no effect, compared to no treatment, on the expression of CD103. The percentage of CD103^+^ DC generated was significantly greater after culture with LL-37, in a concentration-dependent manner (Figure 1(b)), with significantly greater intensity of CD103 expression per cell also observed (Figure 1(c)). These data demonstrated that LL-37 could promote the generation of DC expressing CD103, a marker of the DC subset critical for effective tumor immunotherapy.

A time course of delayed LL-37 exposure showed that LL-37 only enhanced CD103^+^ DC generation when cells were exposed in the first 24 h of culture (Figure 1(d)), indicating modulation of DC differentiation, not simply induced upregulation of CD103 expression on differentiated cells. Furthermore, LL-37 did not alter the total number of cells in the cultures (control cultures mean 1.08 ± 0.2 x 10^6^; LL-37 cultures 1.13 ± 0.29 x 10^6^ per well), nor the percentage of DC generated in the culture (Figure 1(e)). Nevertheless, the proportion of CD103^+^ cells increased over time in culture (Figure 1(f)). Taken together, these data suggested that LL-37 was not extensively expanding a small CD103^+^ precursor population to generate more CD103^+^ DC, but was altering early differentiation of the cells.

We have previously shown that LL-37 can synergize with GM-CSF to enhance ERK1/2 activation.^24^ Therefore, we examined the extent to which use of GM-CSF as the culture growth/differentiation factor in our model was necessary. The use of Flt3-L as a growth factor allows generation of cDC1 cells representative of *in vivo* populations.^25,26^ Use of Flt3-L in control cultures induced a greater proportion of CD103^+^ DC than when GM-CSF was used (Figure 1(g)), as has previously been shown. LL-37 significantly increased the number and proportion of CD103^+^ DC regardless of the growth factor used. However, as GM-CSF generated greater total cell numbers, Flt3-L cultures ultimately resulted in a smaller total number of CD103^+^ DC (Figure 1(h)).

**Figure 2.**
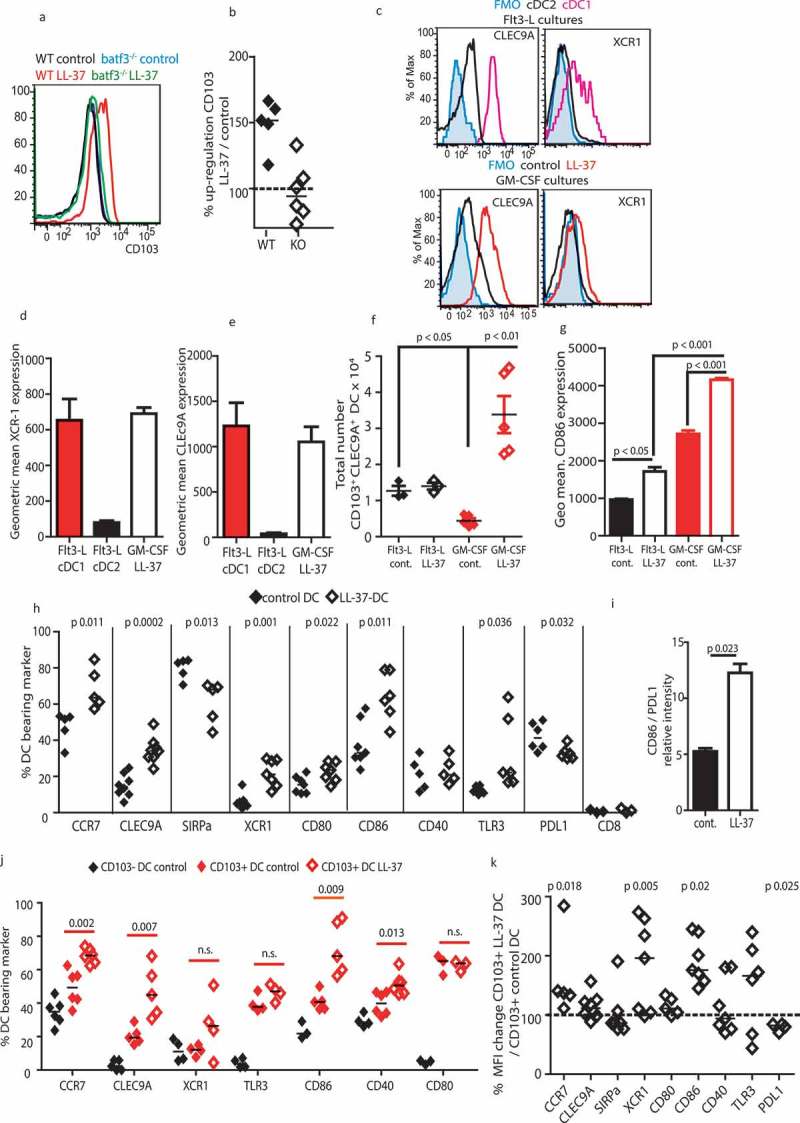
LL-37-DC upregulates markers of cDC1 cells. (a, b) C57Bl6/J (WT) and *Batf3^−/-^* (KO) bone marrow was cultured for 7 days with 20 ng/mL GM-CSF and 10 µM LL-37 or control scrambled peptide. On day 7, CD103 expression was examined by flow cytometry. (c–f) Representative staining showing cDC1 markers on GM-CSF-derived WT LL-37-DC versus Flt3-L-generated DC. (g–k) other markers were expressed on the DC by flow cytometry. Data shown are mean ± standard error or individual data points with line at median. Statistical tests used: (h, i, j, k) two-tailed *t*-test (if necessary on raw data before conversion), *n* = 5–9; (f, g) two-way ANOVA with Bonferroni’s post-test comparing all groups to each other, *n* = 3–5.

CD103^+^ DC populations are dependent upon the transcription factor BATF3 for differentiation and maintenance.^15,22,27^ We examined expression of *Batf3* in WT bone marrow cultures by RT-PCR, and demonstrated that expression was significantly increased, with a mean of 2.8 ± 0.3 fold upregulation following LL-37 exposure. Therefore, next, we examined generation of DC in *Batf3^−/-^* bone marrow cultures. As a consequence of this mutation, few DCs were able to express CD103. However, in these *Batf3^−/-^* cultures, LL-37 was unable to modulate differentiation to enhance generation of CD103^+^ DC populations, demonstrating a requirement for *Batf3* in LL-37-mediated modulation of DC differentiation (Figure 2(a, b)).

### LL-37 enhances differentiation to DC with key features of cDC1 and greater stimulatory potential

Next, LL-37-DC were more fully characterized by examining expression of a panel of other DC markers. Flt3-L cultures were assessed for expression of the cDC1 markers CLEC9A and XCR-1 on cDC1 (as a positive staining control) and cDC2 (as a negative control), and compared to DC generated in GM-CSF in the presence of LL-37 (henceforth referred to as LL-37-DC) or controls (Figure 2(c)). LL-37-DC cultures had levels of CLEC9A and XCR1 comparable to that of cDC1 generated with Flt3-L (Figure 2(c–e)). As has been shown before,^25,26^ Flt3-L generated many more cDC1 cells (mean 12.7 ± 2.3 x 10^3^ in the presence of control peptide) than GM-CSF (4.2 ± 1.8 x 10^3^ with control peptide). LL-37 increased the proportion of CD103^+^ cells in both of these cultures (Figure 1(g)), with consequent decrease in proportion of cDC2 cells (from 82.4 ± 6.4% in Flt3-L control peptide cultures to 75.5 ± 4.3% with LL-37). There was no noticeable impact on pDC populations. However, owing to the increased cell numbers in the GM-CSF cultures, significantly more CD103^+^/CLEC9A^+^cells were generated in GM-CSF cultures with LL-37 compared to Flt3-L cultures (Figure 2(f)). In addition, comparison of these cell populations showed that the co-stimulatory molecule CD86, in particular, was significantly increased on GM-CSF generated DC compared to DC generated in Flt3-L cultures, enhanced to the highest level when exposed to LL-37 (Figure 2(g)). For this reason, because GM-CSF is used in the successful therapy Sipuleucel-T,^4,^^28^ and because of the increase in cell numbers noted in Figure 2(f), we decided that the GM-CSF LL-37 would be more suited to development for immunotherapy, and used these cells for further analysis.

A wider examination of surface markers showed that LL-37-DC displayed a range of characteristics of the naturally occurring subset of cDC1 cells, with a significantly greater proportion expressing CLEC9A, XCR1, CCR7, and TLR3 than in the control population, while significantly fewer LL-37-DC expressed PD-L1 and SIRP-α (Figure 2(h)). This pattern shows the LL-37-mediated upregulation, in a simple *ex-vivo* system, of features representative of cDC1 which are specialized in migrating to lymph nodes and cross-presenting antigen to CD8^+^ T cells.^13,15,29,30^ Migratory and lymphoid-resident cDC1 are distinguished by their expression of CD8.^27,31^ Expression of CD8 on DC was very low and was not affected by LL-37 (Figure 2(h)). It is clear from much other published work that LL-37 is not a necessary growth factor for cDC1, and Figure 2(g) shows that our LL-37-DC are not identical to Flt3-L cDC1; for these reasons we do not call them cDC1 or claim that cDC1 generation *in vivo* depends on LL-37. Instead, these data show that certain features of cDC1 cells can be upregulated in this *ex-vivo* culture model following LL-37 exposure, representing an opportunity for immunotherapeutic DC generation.

In addition to changes in cDC1-related markers, the proportion of DC expressing the co-stimulatory molecule CD86 was dramatically higher in whole LL-37-DC cultures (Figure 2(h)), with a small increase also seen in CD80 expression. Importantly, the ratio of CD86: PD-L1 expression on CD103^+^ DC was therefore dramatically increased in LL-37-DC, compared to control CD103^+^ DC, indicating higher relative co-stimulatory to inhibitory molecule expression and therefore DC more able to efficiently activate T cells (Figure 2(i)).

CD103^+^ LL-37-DC were then compared to control CD103^+^ and CD103^−^ DC. The CD103^+^ LL-37-DC population had a greater proportion of cells expressing CCR7, CLEC9A, and CD86 (Figure 2(j)) than control CD103^+^ DC, and also had even greater per cell expression levels of CCR7, XCR1, and CD86, than control CD103^+^ DC, with significantly less PDL1 expression per cell (Figure 2(k)). CD80 and CD40 were not significantly upregulated (Figure 2(k) and example histograms of co-stimulatory molecules in Figure S1 in Supplementary Material). Therefore, the phenotype of these LL-37-DC was modified, in addition to their increased proportion in culture; these key features of cDC1 were further enhanced by LL-37 when compared to the CD103^+^ DC otherwise generated in culture, with the potential for greater per cell potency during immunotherapy.

**Figure 3.**
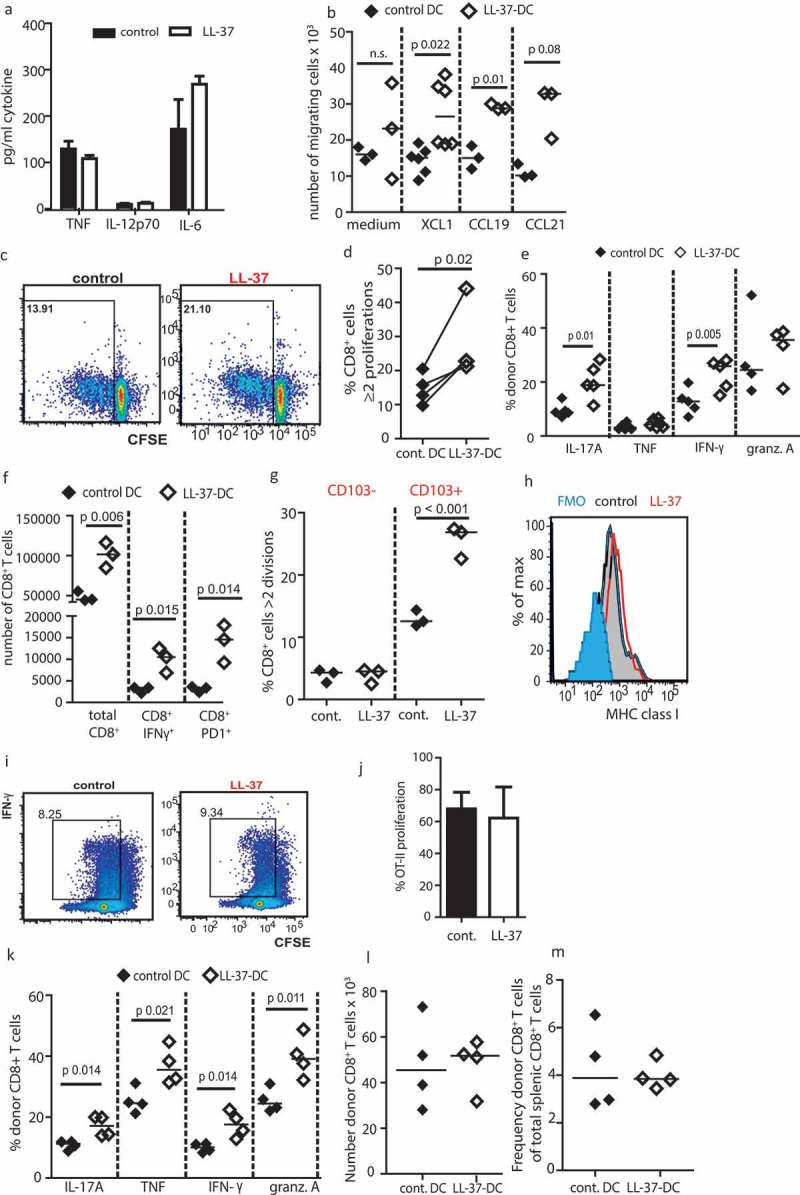
LL-37 DC can cross-present antigen to CD8^+^ T cells. Bone marrow from adult C57Bl6/J mice was cultured for 7 days with 20 ng/mL GM-CSF. (a) production of inflammatory cytokines was assessed by ELISA. (b) Migration of the DC through 5-µM transwells was assessed. (c–e) DC were stimulated with 100 ng/mL Pam3Csk4, poly I:C and ovalbumin peptide for 3 h and their ability to stimulate OT-1 CD8^+^ cell proliferation (c, d) and cytokine production (e) was assessed before the experiment was repeated using whole ovalbumin protein in place of peptide (f). (g) On day 7 of culture DC were sorted by CD103 expression, exposed to whole ovalbumin protein and each population assessed for its ability to induce CD8^+^ T-cell proliferation. (h) On day 7 of DC culture MHC class I expression was assessed by flow cytometry. (i, j) antigen presentation using whole ovalbumin protein was examined with CD4^+^ OT-II cells. (k-m) OT-1 CD8^+^ T cells and stimulated DC were injected into WT mice and subsequently cytokine production (k), number (l) and frequency (m) of donor T cells in the spleen assessed. Data shown are mean ± standard error or individual data points with line at median. Statistical tests used: (b) two-tailed *t*-tests, *n* = 3–5 mice; (d, e, f, k) two-tailed *t*-tests, *n* = 3–6 mice over two experiments; (g) two-way ANOVA with Bonferroni post-test, *n* = 3 mice over two separate experiments.

Given the induction of CD86 expression, to exclude the possibility that LL-37 was simply inducing DC maturation, DC cytokine production was assessed (Figure 3(a)). Cytokine levels were low, and LL-37 had no effect on TNF, IL-12p70 or IL-6 production. These data support our previous study^32^ which demonstrated that LL-37 can induce CD86 on otherwise immature human DC.

### LL-37-DC have enhanced migratory and co-presentation/co-stimulatory function in vitro and in vivo

To determine whether the characteristics of LL-37-DC could be translated for immunotherapy, the functional capabilities of these cells were then assessed. DC optimized for immunotherapy need to be capable of migrating to lymph nodes in a CCR7-dependent manner. Testing DC migration through transwell chambers, toward relevant recombinant chemokines, demonstrated functional competence. Furthermore, LL-37-DC showed significantly enhanced migratory capacity toward XCL-1 and CCL19, compared to control-DC (Figure 3(b)), correlating with their increased expression of the receptors XCR1 and CCR7, respectively.

Another essential feature of DC for immunotherapy is effective priming and activation of cytotoxic T cells. To demonstrate that activated LL-37-DC were functionally capable of presenting antigen and inducing CD8^+^ T-cell proliferation, DC primed with OVA peptide were cultured with transgenic OVA-responding OT-1 CD8^+^ T cells (Figure 3(c, d, e)). These *ex-vivo* cultured LL-37-DC demonstrated enhanced function, generating greater levels of OT-1 CD8^+^ T-cell proliferation and production of IFN-γ and IL-17, than OVA peptide-primed control DC. To confirm these observations in model systems in which DC cross-presentation was required, a series of studies were then conducted *in vitro* and *in vivo*. Firstly, significantly more proliferation of OT-1 CD8^+^ T cells, and greater numbers of IFN-γ^+^ and PD1^+^ cells were also generated by LL-37-DC following processing of whole ovalbumin protein (Figure 3(f)). Importantly, sorting DC by their CD103 expression confirmed that the CD103^+^ cells were the critical cross-presenting population when given whole ovalbumin protein, and showed that, for matched cell numbers, CD103^+^ LL-37-DC induced CD8^+^ T-cell proliferation at a significantly higher rate than CD103^+^ control DC (Figure 3(g)), demonstrating enhanced potency.

To examine the possibility that the increased presentation capacity of LL-37-DC is solely a result of the increased co-stimulation seen in Figure 2(g), two further analyses were then performed. We considered the possibility that LL-37-DC had increased surface expression of MHC class I, resulting in non-specific effects on CD8^+^ T-cell proliferation. MHC class I was quantified on day 7 of DC culture, and was found to be unaffected (Figure 3(h)). Additionally, the impact of LL-37 exposure on antigen presentation to OT-II CD4^+^ T cells was examined. No difference in CD4^+^ T-cell proliferation was observed when ovalbumin protein was presented by LL-37-DC compared to control DC. These data demonstrate that the effects of LL-37 on *ex vivo* cultured DC are specific to DC presenting to CD8^+^ T cells, suggesting that LL-37-mediated modulation of differentiation specifically enhances the cross-presentation capacity of DC *in vitro*. Given the greater co-stimulatory molecule expression on CD103^+^ LL-37-DC, enhanced co-stimulation may also be an important component of the effects demonstrated in Figure 3(f) and/or Figure 3(g). Irrespective, these data suggested the potential for enhanced immunotherapeutic efficacy.

To further characterize this potential, a proof-of-principle *in vivo *examination of the possible therapeutic functionality of these *ex-vivo* generated DC was conducted. Activated LL-37-DC or control-DC (which had been treated with poly I:C and Pam3Csk4) and exposed to ovalbumin protein) were injected into WT mice, 24 h after injection of OT-1 CD8^+^ T cells. Strikingly, the enhancement of cross-presentation to the donor CD8^+^ T cells mediated by LL-37-DC *in vivo* was even greater than *in vitro*, with significantly more splenic CD8^+^ T cells producing granzyme and inflammatory cytokines in response to LL-37-DC than to control-DC (Figure 3(k)). Interestingly however, *in vivo* the total number (Figure 3(l)) and frequency (Figure 3(m)) of donor CD8^+^ T cells found in the spleen were not altered; solely the cytokine production was increased. Taken together, these data demonstrate that LL-37-DC have enhanced potential to cross-present model antigen and upregulate CD8^+^ T-cell proliferation, activation and production of pro-inflammatory cytokines, both *in vitro* and *in vivo*.

### LL-37-DC promote an enhanced CD8^+^ T-cell response and tumor regression when used immunotherapeutically in vivo

**Figure 4.**
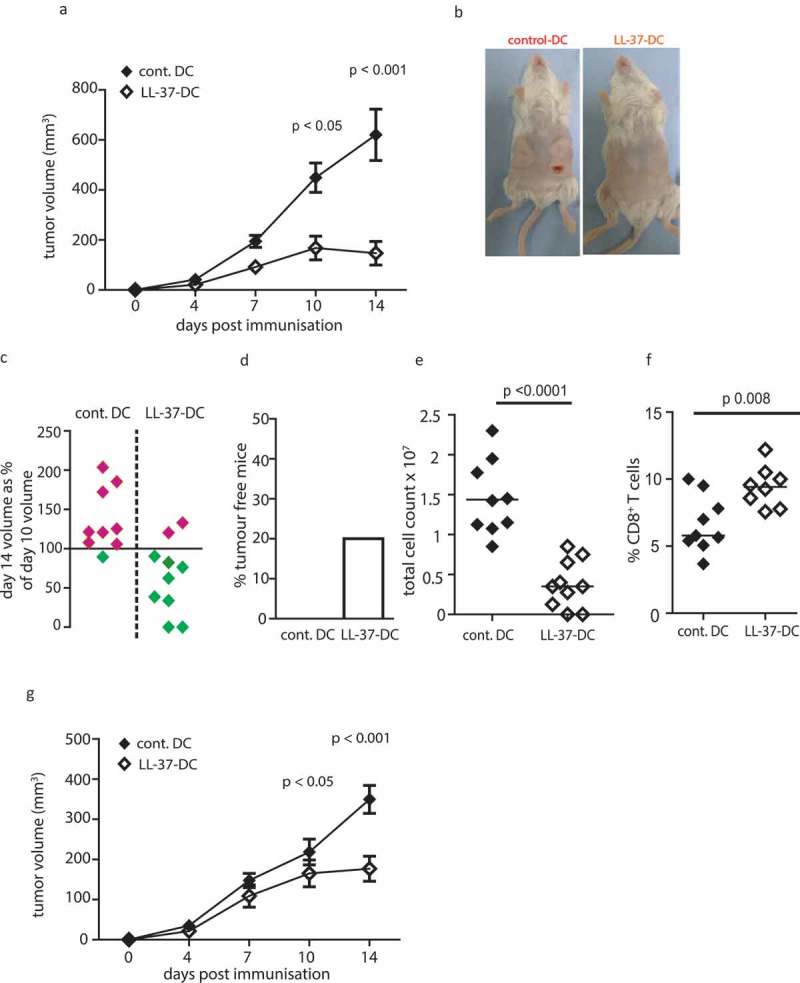
LL-37-DC inducing regression of established tumors. Cells from the squamous cell carcinoma 7.1 cell line were cultured and 0.5 × 10^6^ injected into each flank of FVB mice. On day 4, when tumors were palpable, 0.75 × 10^6^ control-DC or LL-37-DC were injected subcutaneously, centrally in between the tumors. Tumor size was monitored twice weekly (a) until day 14 when mice were culled and examined (b, c, d). Tumors were disaggregated and total viable cell counts performed (e). CD8^+^ T-cell populations were identified by flow cytometry (f). (g) Cells from the SCC 6.2 line were used and tumor growth monitored for 14 days. Data shown are mean ± standard error or individual data points with line at median. Statistical tests used: (a) two-way repeated-measures ANOVA with Bonferroni’s post-test, *n* = 9 tumors treated with control DC and *n* = 10 with LL-37-DC; (e,f) two-tailed *t*-test, *n* = 9 tumors treated with control DC and *n* = 8 with LL-37-DC; (g) two-way repeated -measures ANOVA with Bonferroni's post-test, n=10.

We hypothesized that LL-37 could be used in protocols to increase the yield of immunotherapeutic DC from simple, easily translatable, *ex-vivo* culture systems to generate stronger antitumor immunity. To determine the tumor immunotherapeutic potential of such an approach *in vivo*, a murine squamous cell carcinoma (SCC 7.1) model, which is dependent on cytotoxic CD8^+^ T cells for clearance and resolution,^33^ was used. Four days after tumor cell instillation, tumors had become established and clearly palpable. On this day 7.5 × 10^5^ LL-37-DC or control DC, sorted on the basis of CD11c and MHC II expression, activated with poly I:C and Pam3Csk4 and exposed to sonicated tumor cell antigen, were injected subcutaneously. Injections were applied centrally, between the two tumor injection sites. Mice given control DC experienced uncontrolled growth of tumors in all cases, with ulceration in 40%, requiring termination of the study on day 14 for welfare purposes. Strikingly, mice given LL-37-DC showed significantly less tumor growth (Figure 4(a); individual mice shown in Figure S2(a) in Supplementary Material). Complete clearance of two LL-37-DC-treated tumors was observed, with regression in six others (Figure 4(b, c, d)).

Total cell counts of disaggregated tumors were significantly greater in control DC-treated mice than those from LL-37-DC-treated mice, further demonstrating tumor regression (Figure 4(e)). Despite this, flow-cytometric analyses showed that the regressing tumors remaining in LL-37-DC-treated mice contained a significantly higher percentage of CD8^+^ T cells than the controls (Figure 4(f)). The significantly lower total cell counts in LL-37-DC treated tumors meant that overall total intratumoral CD8^+^ T-cell numbers were, however slightly lower in these mice (Figure S2(c) in Supplementary Material). Finally, to confirm these data, an alternative genetically distinct tumor model (SCC 6.2) was also used, also demonstrating significantly enhanced control of tumor growth by LL-37-DC therapy, in comparison to control DC (Figure 4(g), individual mice shown in Figure S2(b) in Supplementary Material).

**Figure 5.**
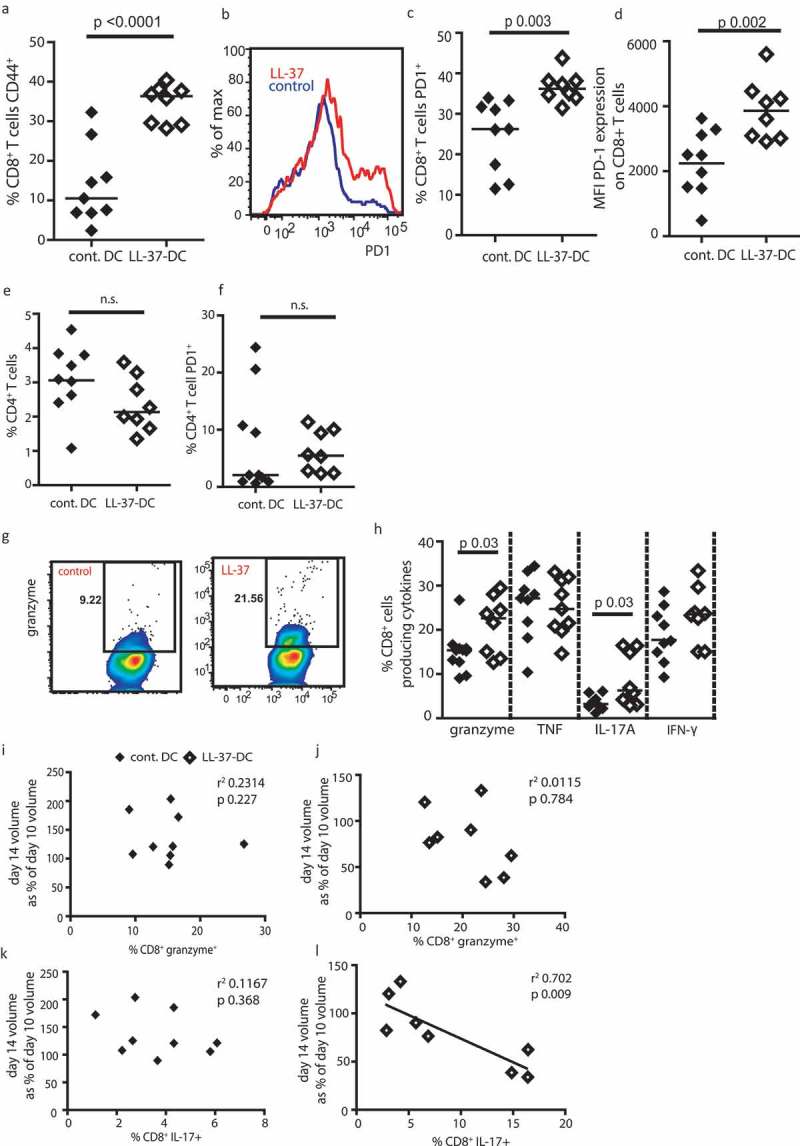
Tumors treated with LL-37-DC contain granzyme^+^ PD-1^+^ CD8^+^ T cells. Cells from the squamous cell carcinoma 7.1 cell line were cultured and 0.5 × 10^6^ injected into each flank of FVB mice. On day 4, when tumors were palpable, 0.75 × 10^6^ control-DC or LL-37-DC were injected subcutaneously. (a-h) Tumors were disaggregated and T-cell populations assessed by flow cytometry. (i - l) correlation analyses were performed comparing cytokine production to volume alterations in tumors. Data are shown as individual data points with line at median. Statistical tests used: a, c, d, e, f, h) two-tailed *t*-tests, *n* = 9 tumors treated with control DC and *n* = 8 with LL-37-DC; i, j, k, l) Pearson correlation analysis, *n* = 9 tumors treated with control DC and *n* = 8 with LL-37-DC.

Next, the intratumoral T-cell phenotype from disaggregated tumors was examined using flow-cytometry analysis. CD8^+^ T cells in mice given LL-37-DC were more activated, with a striking increase in CD44 expression (Figure 5(a)). LL-37-DC also induced a significantly higher frequency of CD8^+^ T cells expressing PD-1 in the tumor (Figure 5(b, c)), of critical functional importance on tumor-infiltrating T cells. Furthermore, the expression intensity of PD1 per cell was significantly increased on CD8^+^ T cells generated by LL-37-DC (Figure 5(d)). Interestingly, and reflecting the *in vitro* OT-II cell experiment *in vitro*, CD4^+^ T cells were not significantly increased in proportion (Figure 5(e)) or PD1 expression (Figure 5(f)) following LL-37-DC treatment compared to control DC.

The intratumoral CD8^+^ T cells from the LL-37-DC-treated mice also produced significantly more granzyme A and IL-17A (Figure 5(g, h)), than those from control DC treated animals, demonstrating effector function. Correlation analyses showed that greater expression of granzyme A by intratumoral CD8^+^ T cells was not correlated with the regression of the tumor (day 14 volume/day 10 volume) (Figuer 5(i, j)). However, IL-17A expression was significantly correlated with tumor regression in LL-37-DC treated mice, but not controls (Figure 5(k, l)).

Taken together, these data demonstrate that the characteristics of LL-37-DC which indicated the potential for enhanced immunotherapeutic functionality do translate to increased efficacy in *in vivo* tumor models. Thus, the simple *ex-vivo* culture of DC in the presence of LL-37 is a novel, translatable mechanism to rapidly generate large numbers of DC with an enhanced profile of suitability for immunotherapy, capable of generating CD8^+^ T cells with potent antitumor potential *in vivo*.

### LL-37 enhances differentiation of human DC with key features of cDC1

**Figure 6.**
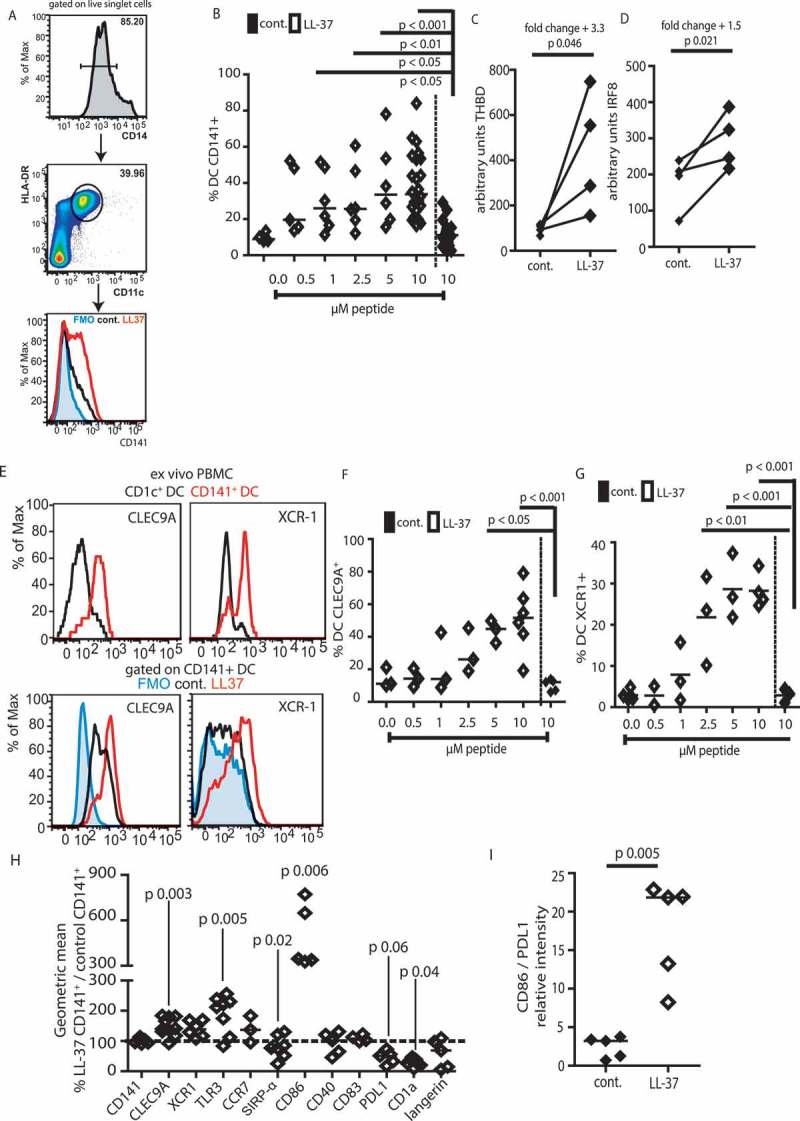
Human DC exposed to LL-37 upregulate CD141, CLEC9A, and XCR1. Monocytes were isolated from blood of healthy volunteers and incubated for 7 days with GM-CSF, IL-4 and 10 µM LL-37 or control peptide. On day 7, DC were identified as in (a) and flow cytometry (b) used to assess expression of CD141. (c–d) 24 h after incubation with LL-37 RNA was isolated from CD11c^+^ HLA-DR^+^ cells and expression of *THBD* and *IRF8* examined. (e) Staining of cDC1 markers on LL-37-DC was compared to CD141^+^ DC isolated from blood. (f–i) other markers were examined on day 7 of culture. Data are shown as individual data points with line at median. Statistical tests used: (b, f, g) one-way ANOVA with Dunnett’s post-test comparing all to control, *n* = 3–24 donors; (c, d, h, i) two-tailed *t*-test, if necessary on raw data before conversion, *n* = 4–8 donors.

Finally, to initiate examination of translational capacity to human DC immunotherapy, we sought to determine whether LL-37 induced similar alterations in human DC. We aimed to firstly examine effects in a culture system of maximal translatable simplicity, without pre-sorting cells. Therefore, human PBMC from healthy donors were cultured with GM-CSF and IL-4, in the presence or absence of LL-37 or control scrambled peptide. As observed in the mouse cultures, total cell numbers were unaffected by LL-37 treatment (control cultures mean 7.2 ± 0.8 x 10^5^, LL-37 cultures 7.4 ± 1.4 x 10^5^ cells per well). At day 7 of culture, phenotypic flow-cytometric assessment was conducted, identifying DC according to the gating strategy shown in Figure 6(a). In a parallel to the murine data, DC cultured in the presence of LL-37 strongly upregulated CD141, a marker of a DC sub-population considered the human equivalent to murine cDC1,^12^ in a concentration-dependent manner (Figure 6(b)). Culture with LL-37 generated 38.7 ± 3.5% CD141^+^ DC, with up to 80% CD141^+^ DC in one donor, amplified from 11.5 ± 1.3% CD141^+^ DC with control peptide; a large increase compared to naturally-occurring populations or published *ex vivo* strategies for generating these cells.^30^ Having demonstrated that early exposure to LL-37 was essential in murine cultures to modulate DC differentiation, RNA was isolated from CD11c^+^ HLA-DR^+^ cells at 24 h after incubation with LL-37 and analyzed for transcription of markers relating to generation of CD141^+^ DC (Figure 6(c, d)). Both *THBD* (CD141) and *IRF8* were significantly upregulated following LL-37 exposure, providing strong support to the flow-cytometric characterization.

CD141 is not a specific marker of cDC1,^34–36^ so key markers of functionally-appropriate immunotherapeutic DC (XCR1 and CLEC9A) were also examined. To validate our flow-cytometry staining, we analyzed the small *ex vivo* DC population isolated from peripheral human blood and compared staining intensities with our cultured cells (Figure 6(e)). XCR-1 expression was lower in our LL-37-DC than in *ex vivo* CD141^+^ DC but was clearly upregulated over control culture-generated cells, which showed no expression. CLEC9A was expressed on the small number of control culture-generated CD141^+^ cells at an intensity similar to that seen in the human blood isolated cells, and at a higher level in LL-37-DC (Figure 6(e)). The proportion of DC expressing these markers in culture was upregulated in a concentration-dependent manner by LL-37 treatment on whole DC cultures (Figure 6(f, g)).

In addition, CD141^+^ LL-37-DC also expressed higher levels of CLEC9A, XCR1, and TLR3 on a per cell basis (Figure 6(h)), in addition to dramatically greater levels of CD86 and reduced PDL1, showing that LL-37 not only promoted generation of more of these cells, but that they also had enhanced expression of markers characteristic of the naturally-occurring DC with cross-presenting DC. Importantly, mirroring the murine studies, the ratio of CD86: PDL1 expression on CD141^+^ LL-37-DC was significantly increased compared to controls (Figure 6(i)), demonstrating enhanced stimulatory potential.

We have previously shown that LL-37 can enhance co-stimulatory molecule expression on otherwise immature DC.^32^ However, to further establish that the changes described in Figure 6 are not simply a consequence of maturation, monocyte-derived DC cultures were exposed to either LL-37, LPS, or polyI:C and examined for altered expression of CLEC9A and XCR-1 by flow cytometry. Whereas LL-37 induced upregulation of CLEC9A and XCR-1 on the DC (Figure S3 in Supplementary Material), the other treatments did not. These data confirm that (i) the effects of LL-37 on DC differentiation are not a result of simple maturation and (ii) LL-37 is unusual in its ability to modify the expression of co-stimulatory molecules and features of cross-presenting DC in a simple human DC culture system.

**Figure 7.**
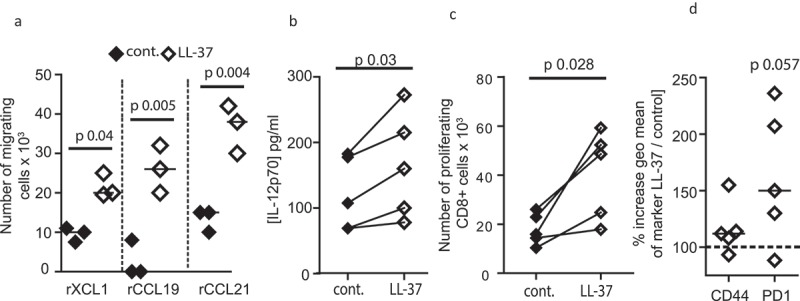
Human LL-37-DC induce CD8^+^ T-cell proliferation. Monocytes were isolated from blood of healthy volunteers and incubated for 7 days with GM-CSF, IL-4 and 10 µM LL-37 or control peptide. (a) On day 7, CD11c^+^ HLA-DR^+^ cells were sorted and migration through 5 µM transwells toward recombinant chemokines assessed; (b) sorted DC were stimulated with polyI:C and IL-12p70 production assessed by ELISA. (c, d) sorted DC were incubated with Epstein Barr Virus peptides and poly I:C before incubation with CFSE-labeled autologous CD8^+^ T cells. 60 h later T-cell proliferation (c) and activation (d) were assessed. Data are shown as individual data points with line at median. Statistical tests used: (a–d) paired two-tailed *t*-tests, if necessary on raw data before conversion, *n* = 3–5 donors.

Next, the functional significance of LL-37-modulated differentiation was assessed. Human LL-37-DC demonstrated significantly increased chemotaxis to XCL1, CCL19, and CCL21 compared to control DC (Figure 7(a)), mirroring the results with murine cells and correlating with the changes to chemokine receptor expression. In addition, LL-37-DC, stimulated with 100 ng/mL polyI:C, produced greater levels of IL-12p70 release than control DC (Figure 7(b)), in keeping with our previously demonstrated enhanced IL-12 production phenotype for activated human LL-37-DC.^32^ This is key, as IL-12 production by DC is critical to inducing a strong antitumor CD8^+^ T-cell response.^37^

Finally, to examine whether human LL-37-DC could induce enhanced responses in autologous CD8^+^ T cells, labeled human T cells were co-incubated with DC primed with poly I:C and Epstein Barr Virus peptides. LL-37-DC induced significantly greater CD8^+^ T-cell proliferation (Figure 7(c)) than control DC, with greatly increased expression of activation markers, including PD-1, on these T cells (Figure 7(d)).

These data demonstrate that, as an initial proof-of principle, human LL-37-DC are primed to induce enhanced activation of CD8^+^ T cells, justifying future evaluation of the translatable potential for the use of LL-37 in the culture of immunotherapeutic DC development toward novel human tumor therapies.

## Discussion

We show a novel role for the antimicrobial host defense peptide cathelicidin LL-37 in reprogramming DC differentiation toward a CD103^+^/CD141^+^ phenotype that exhibits significantly enhanced antitumor activity. Specifically, DCs differentiated in the presence of LL-37 showed increased expression of (i) co-stimulatory molecules, (ii) markers associated with antigen cross-presentation, (iii) inflammatory cytokines, and (iv) chemokine receptors that facilitate migration to lymph nodes. In addition to greatly enhanced yield of these DC in culture, which is a current priority for DC immunotherapy approaches, these *ex-vivo* generated LL-37-DC had further enhancement of the key features required for immunotherapy when compared to CD103^+^/CD141^+^ DC generated using standard culture methodology. Key functional characterization demonstrated that CD103^+^ LL-37-DCs elicited an enhanced cytotoxic T-cell response *in vitro* and *in vivo* with enhanced CD8^+^ T-cell activation, PD-1 expression, cytokine and granzyme production. Importantly, when used in a model of DC immunotherapy of solid tumors, LL-37-DC induced CD8^+^ T-cell infiltration into tumors and resulted in tumor regression. Critically, these effects of LL-37 were not restricted to murine DCs, with human LL-37-DC showing differentiation to a CD141^+^ phenotype, with a similarly enhanced expression of key molecules required to induce antitumor immunity, and enhanced chemotactic and cross-presentation function *in vitro*. Therefore, LL-37 directs the upregulation of markers of the cDC1 subset of cells which are known to be useful for immunotherapy. We do not propose that addition of LL-37 directs the generation of cells identical to the naturally-occurring cDC1 found *in vivo*, or that LL-37 is required *in vivo* for cDC1 differentiation; rather that key features of cDC1 which LL-37 exposure can enhance in *ex-vivo* cultured DC make this an attractive, cheap and simple way of boosting immunotherapy-optimized cell populations.

The drive to develop DC-based therapy as a cancer treatment has resulted in a wide range of protocols varying in the method of cell isolation, maturation cocktail, time of culture, site and frequency of administration (reviewed in^38^). This has led to a multitude of options and so far no consensus has been reached on the optimal method. Whether to use natural DC, which have superior cross-presentation and cytokine producing capabilities but are rare *in vivo*, or to use monocyte-derived DC boosted *ex vivo*, is a key question in immunotherapy. So far, monocyte-derived DC are more frequently used, and some success is noted^4^ but there are clear roadblocks with respect to appropriate DC chemotaxis to lymph nodes and with the low numbers of the suitable DC population, required to prime antitumor CD8^+^ responses, generated using standard methodologies.^3,39,40^ We propose that use of LL-37 can substantially address these critical issues in monocyte-derived DC cultures for immunotherapies.

Conventional differentiation cocktails, including PGE-2, are clearly suboptimal due to impaired induction of IL-12p70 expression and their propensity to induce T regulatory cells.^41^ A more recent method using TNF, IL-1β, poly I:C, IFN-α, and IFN-γ to generate αDC1 cultures has overcome some of these issues, resulting in cells that produce IL-12, migrate, activate CD8^+^ T cells and are safe *in vivo*.^42,43^ However, it is not clear to what extent this method promotes enrichment for CD103^+^/CD141^+^ DCs (or functional equivalents), which have been reported to be the antigen presenting cells capable of transporting intact antigens to lymph nodes and activating tumor-specific CD8 T-cell-mediated immune responses.^14,18,20^ Systemic administration of Flt3-L followed by intratumoral poly I:C injections^20^ has been shown to increase the number of CD103^+^ DCs in tumors and their expression of co-stimulatory molecules, resulting in enhanced tumor responses to anti-PDL1 immune checkpoint inhibitors and BRAF inhibitors. We show that LL-37 affects DC phenotype when added to either GM-CSF or Flt3-L as a maturation cocktail but generated much larger quantities of more potent immunotherapeutic DC when applied to GM-CSF cultures. Importantly, our approach is successful using simple preparations of cells isolated from blood, without the need for expensive cell sorting. Therefore, in contrast to the increasingly complex and expensive approaches being developed for generation of DCs capable of stimulating antitumor immune responses, we propose that the addition of LL-37 to standard maturation cocktails represents a simple, cost-effective, and readily scalable approach that can be easily developed for clinical translation.

None of the changes LL-37 induces in DC are unique, but we believe it is the combination of effects which makes LL-37-DC attractive for immunotherapy. One simple, cheap, easily scalable culture system which can simultaneously upregulate cross-presentation and co-stimulatory function, migration toward lymph nodes, IL-12 production, and PD-1 expression on responding T cells, is hugely attractive for translation. In addition to increased numbers generated, LL-37 induced significantly higher CD86 over Flt3-L cDC1, suggesting use of these DC, although not classical cDC1, may lead to significant improvements to immunotherapeutic protocols.

Co-stimulation is critical to a strong antitumor T-cell response, a prerequisite for successful immunotherapy.^20^ LL-37 induces upregulation of CD86 on DC very strongly, and likely contributes to the enhanced cross-presentation capacity observed, giving LL-37-DC very exciting therapeutic potential. Future studies will determine the relative contributions of increased CD86 expression and the possibility of modified cross-presentation processes to the enhanced functioning of these DC, using assays which are not dependent on co-stimulation. Additionally, we show that CCR7 was upregulated on both mouse and human DC following LL-37 exposure. CCR7 is expressed on intratumoral cDC1 and strongly correlates with infiltration of T cells into human tumors.^18^ This may therefore be a key driver of the antitumoral T-cell responses we see in the murine SCC model.

Using two murine models of SCC we show that LL-37-DC can induce a robust antitumor response and even complete tumor regression when compared to DCs generated using standard approaches. Underpinning this response, we observed increased infiltration of activated CD8^+^ T-cells into SCC tumors following LL-37 DC treatment. Not only did more tumor infiltrating CD8^+^ T-cells in LL-37DC treated tumors express the activation markers CD44 and PD1, but we also observed an increase in the expression level of PD1 on these CD8^+^ T-cells. PD1 is frequently used as a marker of T-cell exhaustion. However, it is primarily a marker of T-cell activation, and the level of PD1 expression is related to the strength of TCR signaling and ultimately CD8^+^ T-cell functional avidity.^44^ Furthermore, PD1 expression has been shown to mark the tumor reactive CD8^+^ T-cell population,^45^ and its expression on infiltrating T cells has been correlated with favorable clinical outcomes.^46^ Therefore, these findings imply that DCs differentiated in the presence of LL-37 induce a robust and tumor-specific CD8^+^ T-cell response that, at least in pre-clinical murine models of cancer, can significantly improve outcome. Clearly, increased expression of PD1 will also render CD8^+^ T-cells more susceptible to negative signaling, suggesting that use of anti-PD1 immune checkpoint inhibitors in combination with LL-37 DCs may further enhance therapeutic efficacy, representing an exciting avenue for further research development. Finally, a new avenue of research aims to generate DC by methods other than traditional culture; by using CD34^+^ stem cells to generate the necessary DC subsets in large volume^47,48^ . The impact of LL-37 in these culture systems will be of great interest.

Overall, these findings provide good rationale to support further pre-clinical and clinical development of LL-37 as an important co-factor for maturation and activation of DCs for use in anticancer immunotherapy.

## Methods

### Mice

C57Bl/6JOlaHsd mice were bred in house in individually ventilated cages and maintained under specific pathogen-free conditions. Both male and female mice were used, between 6–12 weeks of age. Spleens from *Rag-1^−/−^* OT-I CD45.1^+/+^ mice, maintained in individually ventilated cages under specific pathogen-free conditions, were a kind gift from Professor Rose Zamoyska, University of Edinburgh. Spleens from Rag1^−/-^ OT-II mice, maintained in individually ventilated cages under specific pathogen-free conditions, were a kind gift from Professor Stephen Anderton, University of Edinburgh. These were transported on ice and used within 2 h after cull.

Femurs from *Batf3^−/-^* mice, maintained in individually ventilated cages under specific pathogen-free conditions, and backcrossed for 10 generations onto in-house C57Bl6/J mice, were a kind gift from Professor Andrew Macdonald, MCCIR, University of Manchester; these were transported on ice and used 24 h after cull.

Cancer studies were performed on female FVB/NJ mice, 6–7 weeks old, purchased from Charles River UK and housed in individually ventilated cages under specific pathogen-free conditions.

### Isolation and culture of mouse bone marrow-derived dendritic cells

Femurs were removed from 6 to 12 week old C57Bl/6J mice and the marrow flushed out with complete medium (RPMI, 10% fetal calf serum, 10 units/mL penicillin, 10 μg/mL streptomycin and 2 mM L-glutamine, all supplied by Gibco, ThermoFisher UK). Red blood cells were lysed (RBC lysis buffer, Biolegend UK) and a single cell suspension made by passing through a 19G needle. 1 × 10^6^ cells were plated per well of a 24-well plate. After 1 h incubation at 37°C, non-adherent cells were removed and complete RPMI containing 20 ng/mL GM-CSF or 100 ng/mL Flt-3L (both R&D Systems) was added. Time from removal of femur to culture was typically 3 h.

Cells were treated with LL-37 or scrambled control peptide (both custom synthesized by Almac, Penicuik, Scotland) at a range of doses. On day 4 of culture all medium was gently removed from the wells and complete medium with GM-CSF or Flt3-L plus peptides replaced.

### Peptide synthesis

LL-37 (LLGDFFRKSKEKIGKEFKRIVQRIKDFLRNLVPRTES) and scrambled LL-37 (RSLEGTDRFPFVRLKNSRKLEFKDIKGIKREQFVKIL) were custom synthesized (Almac, Penicuik, Scotland) using Fmoc solid phase synthesis and reversed phase HPLC purification. Peptide identity was confirmed by electrospray mass spectrometry, purity (>95% area) by RP-HPLC and net peptide content determined by amino acid analysis. Lyophilized peptides were reconstituted in endotoxin free water at 5 mg/mL stock concentration and determined to be endotoxin-free using a Limulus Amebocyte Lysate Chromogenic Endotoxin Quantitation Kit (Thermo Scientific, UK).

### Flow cytometry

On day 7, cells were stained for 30 min at 4°C in the dark (with the exception of anti-CCR7, which was stained at 37°C), and samples were fixed with 1% paraformaldehyde or run unfixed. Samples were analyzed using a Fortessa LSR II (BS Biosciences) and FlowJo software (Treestar). Intracellular cytokines were assessed by incubating cells for 4 h with Cell Stimulation Cocktail containing brefeldin A (ebioscience UK). Subsequently cells were stained for surface markers as above then washed, fixed and permeabilised with the BD fixation/permeabilisation kit (BD Biosciences UK). Intracellular cytokines were stained with antibodies in BD perm wash buffer. Cells were re-suspended in PBS and run on the Fortessa LSR II within 24 h.

### Antibodies used

## Mouse

CD45.1 (clone A20, Biolegend cat 110718); CD86 (PO3.1, Ebioscience 12–0861-82); CD86 (GL-1, Biolegend 105035); CD103 (2E7, Biolegend 121422); CD11c (N418, Biolegend 117339); MHC II (M5/114.15.2, Biolegend 107623); PDCA-1 (927, Biolegend 127015); F4/80 (BM8, Ebioscience 47–4801-80); CD8 (53–6.7, BD Biosciences 563234); CD8 (53–6.7, Ebioscience 45–0081-82); CCR7 (4B12, Biolegend, 120112); CLEC9A (7H11, Biolegend UK, 143504); XCR1 (ZET, Biolegend, 148205); TLR3 (11F8, Biolegend, 141905); CD80 (16-10A1, Biolegend, 104705); CD40 (3–23, Biolegend, 124621); SIRPa (p84, Biolegend, 144006); PDL1 (10F.9G2, Biolegend, 124312); CD3 (145-2C11, Biolegend, 100227); CD44 (IM7, Biolegend, 103016); CD44 (IM7, Biolegend, 103039); PD1 (29F.1A12, Biolegend, 135016); IL17A (TC11-18H10.1, Biolegend, 506925); IFN-γ (XMG1.2, Biolegend, 505825); TNF (Mab11, Biolegend, 506313); TNF (MP6-XT26, Ebioscience, 48–7321-82); Granzyme A (3G8.5, Biolegend, 149704); MHC class I (28–14-8, Biolegend, 114507).

## Human

CD86 (clone IT2.2, Biolegend UK, cat 305413); CD86 (IT2.2, Ebioscience UK, 12–0869-42); CD141 (M80, Biolegend, 344112); CD11c (3.9, Biolegend, 301638); HLADR (L243, Ebioscience, 47–9956-42); CD14 (HCD14, Biolegend, 325610); CD14 (63D3, Biolegend, 367118); CLEC9A (8F9, Biolegend, 353804); XCR1 (1097A, RnD Systems, FAB857F); XCR1 (S15046E, Biolegend, 372611); TLR3 (TLR-104, Biolegend, 315005); CCR7 (G043H7, Biolegend, 353207); SIRPa (SE5A5, Biolegend, 323809); CD40 (5C3, Biolegend, 334321); CD83 (HB15e, Biolegend, 305312); PDL1 (29E.2A3, Biolegend, 329717); CD1a (HI149, Biolegend, 300122); Langerin (10E2, Biolegend, 352201); CD8 (OKT8, Ebioscience, 17–0086-42); CD44 (IM7, Ebioscience, 48–0441-82); PD1 (EH12.2H7, Biolegend, 329906).

### Murine cross-presentation assay

DCs were generated as described. On day 7, CD11c^+^ MHC II^+^ cells were isolated by FACS sorting, plated in 96 well U-bottom plates and stimulated with 5 μg/mL ovalbumin peptide 257–264 for 3 h (Sigma) or 1 µg/mL whole ovalbumin protein (Sigma) overnight, plus 100 ng/mL polyI:C and 100 ng/mL Pam3CSK4 (Invivogen). CD8^+^ T cells from spleens of OT-1 mice were isolated by magnetic separation using anti-CD8 beads (Miltenyi Biotec) and CFSE-labeled for 20 min at 37°C (Life Technologies). T cells were layered on top of DC at a 1:8 DC: T ratio. After 60 h proliferation and cytokine production was assessed by flow cytometry as before.

In some experiments, cross-presentation was assessed *in vivo*. 2 × 10^7^ CFSE-labeled OT-1 CD8^+^ T cells were injected intravenously into C57Bl6/J mice. CD11c^+^ MHC II^+^ cells were isolated by FACS sorting, plated in 96 well U-bottom plates and stimulated with 1 µg/mL whole ovalbumin protein, 100 ng/mL polyI:C and 100 ng/mL Pam3CSK4 overnight, then injected subcutaneously in the flank. 60 h later spleens were removed and T-cell proliferation and cytokine production assessed by flow cytometry.

### Murine antigen-presentation assay

DC were generated as before. On day 7, CD11c^+^ MHC II^+^ cells were isolated by FACS sorting, plated in 96 well U-bottom plates and stimulated with 1 µg/mL whole ovalbumin protein (Sigma) overnight, plus 100 ng/mL polyI:C and 100 ng/mL Pam3CSK4 (Invivogen). CD4^+^ T cells from spleens of OT-II mice were isolated by magnetic separation using anti-CD4 beads (Miltenyi Biotec) and CFSE-labeled for 20 min at 37°C (Life Technologies). T cells were layered on top of DC at a 1:8 DC: T ratio. After 60 h proliferation and cytokine production was assessed by flow cytometry as before.

### Migration assay

3 x 10^5^ DC, in 100 µl complete medium, were plated in the top well of a 5 µM-pore transwell (Corning). The bottom wells contained 600 μl complete medium, with or without 100 ng/mL recombinant XCL1, CCL19 or CCL21 (RnD Systems, Abingdon, UK). Plates were incubated at 37°C for 3 h before medium was removed from the bottom wells and cell counts performed on a hemocytometer.

### Subcutaneous tumor growth and treatment

SCC cells were generated as previously described, from carcinomas arising on FVB/NJ mice.^49^ Cell lines were authenticated using western blot analysis and were pathogen tested using the Impact III test (Idex Research). Lines were tested for mycoplasma at least every 2–3 months and have never tested positive. Cell lines are passaged for less than three months after thawing.

SCC7.1 and SCC6.2 cells were cultured in Glasgow minimum essential medium (Sigma, UK) supplemented with 10% fetal calf serum, 2 mM L-glutamine, non-essential amino acids, sodium pyruvate and MEM vitamins (all Life Technologies, UK) at 37 °C, 5% CO2. To prepare SCC cells for injection into mice they were washed with PBS, re-suspended in normal growth media and pelleted by centrifugation at 234xg for 5 min. Cell pellets were re-suspended in HBSS at a concentration of 0.5 × 10^6^ cells per 100 mL.

Tumors were established using bilateral subcutaneous injection of 0.5 × 10^6^ SCC 7.1 or SCC6.2 cells into each flank of 6–8 week old female FVB/NJ mice (Charles River UK). Tumors were monitored and measured with calipers. On day 4, once tumors were palpable and visible, mice were treated. Control- or LL-37-DC were generated from FVB bone marrow for 7 days, flow-sorted by CD11c and MHC II expression, and incubated for 3 h with sonicated tumor antigen, 100 ng/mL polyI:C and 100 ng/mL Pam3CSK4. Cells were washed well and 0.75 × 10^6^ injected subcutaneously centrally between the two tumors. Mice were monitored until day 14 then culled and tumors removed. Tumors were disaggregated by mincing with scissors then incubating for 1 h at 37°C with 20 µg/mL DNase I and 2 mg/mL Collagenase D (both Roche Products Ltd), with shaking at 80 rpm. Red blood cells were lysed and cell suspensions passed through a 70 µm sieve before being stained for flow cytometry as before.

### Isolation of PBMC and neutrophils and generation of human DC

PBMCs were isolated from healthy adult volunteers by dextran sedimentation and Percoll gradient as detailed in.^50^ Volunteers were male or female, between 21 and 55 years of age. 1 × 10^6^ PBMC were plated per well in a 24-well plate in complete medium. One hour later non-adherent cells were removed and complete RPMI containing 100 ng/mL GMCSF and 20 ng/mL IL-4 (both R&D Systems) was added in addition to LL-37 or control scrambled peptide. On day 7 cells were removed for flow cytometry or migration assays, as before.

### Analysis of gene expression in human LL-37-DC

DCs were isolated and plated as above from four human donors. 24 h after plating in the presence or absence of LL-37, CD11c^+^ MHC II^+^ cells were isolated by cell sorting and RNA extracted using an RNeasy micro kit (Qiagen). 100 ng RNA was loaded per sample onto a Nanostring human inflammation chip (Nanostring Technologies) and run on an nCounter analyzer.

### Human cross-presentation assay

On day 7 of culture DC were flow sorted based on high CD11c and HLA-DR expression, and incubated with 100 ng/mL poly I:C and 100 ng PepTivator EBV BRLF-1 per 1 × 10^6^ cells (PepTivator is a pool of peptides covering the complete Epstein Barr Virus protein sequence, Miltenyi Biotec) for 3 h. The same donors were bled again and T cells isolated by magnetic sorting with anti-CD3 beads (Miltenyi) before being CFSE labeled for 20 min at 37°C (Life technologies). DC were washed well and co-cultures set up at a 1:8 DC: T-cell ratio. After 60 h proliferation and activation of the T cells were assessed by flow cytometry.

## ELISA

Human IL-12p70 and mouse IL-6, TNF and IL-12p70 concentrations were determined with eBioscience UK ELISA kits, used according to manufacturers’ guidelines.

### Statistical analysis

All data shown are expressed as mean ± SEM or individual data points with line at median. Multiple groups were compared by one- or two-way analysis of variance tests with either Bonferroni or Dunnett post-tests as appropriate. Two groups were compared with Student’s *t*-test. Analysis was performed with GraphPad Prism software. A minimum of three mice or donors was used for *in vitro* experiments, in individually performed experiments. Details of sample sizes are included in all figure legends.

*In vivo* tumors were induced by one experimenter who was blind to treatment groups; a second experimenter injected the DC. Sample sizes for these experiments were based on knowledge of the model from previous studies.^49^

### Study approval

Mouse breeding and experimental work was carried out in accordance with the Animal (Scientific Procedures) Act 1986, PPL number 70/8884, and under the supervision of the University of Edinburgh Ethical Review Committee. All researchers were accredited by the Home Office, UK. Cancer studies were performed on PPL number 60/4248 under the supervision of the University of Edinburgh Ethical Review Committee.

Peripheral blood cells were isolated from healthy adult human volunteers under Ethical Review (AMREC Reference number 15-HV-013) under the project number CIRBRP003; this included written informed consent.
