# Supplementary figures and images for: Exposure to the antimicrobial peptide LL-37 produces dendritic cells optimized for immunotherapy
Source: Oncoimmunology. 2019 May 1;8(8):1608106. doi: 10.1080/2162402X.2019.1608106 (PMC6682359; doi:10.1080/2162402X.2019.1608106)

Supplementary Figure 3

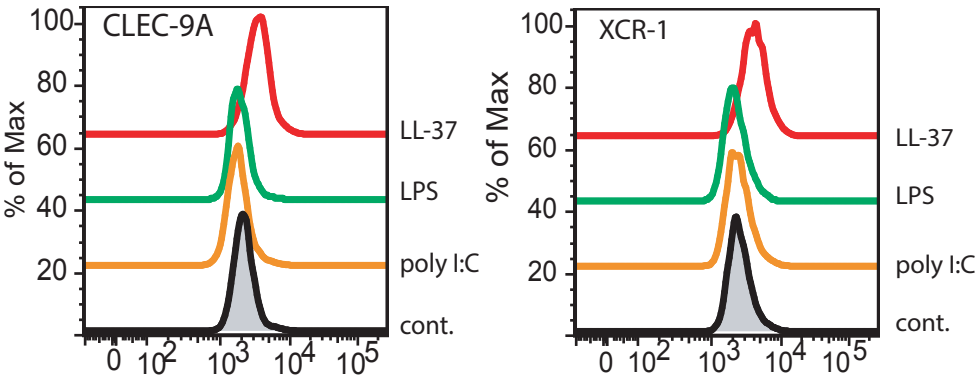

Supplement: Supplemental Material [file koni-08-08-1608106-s001.pdf]

Supplementary Figure 2

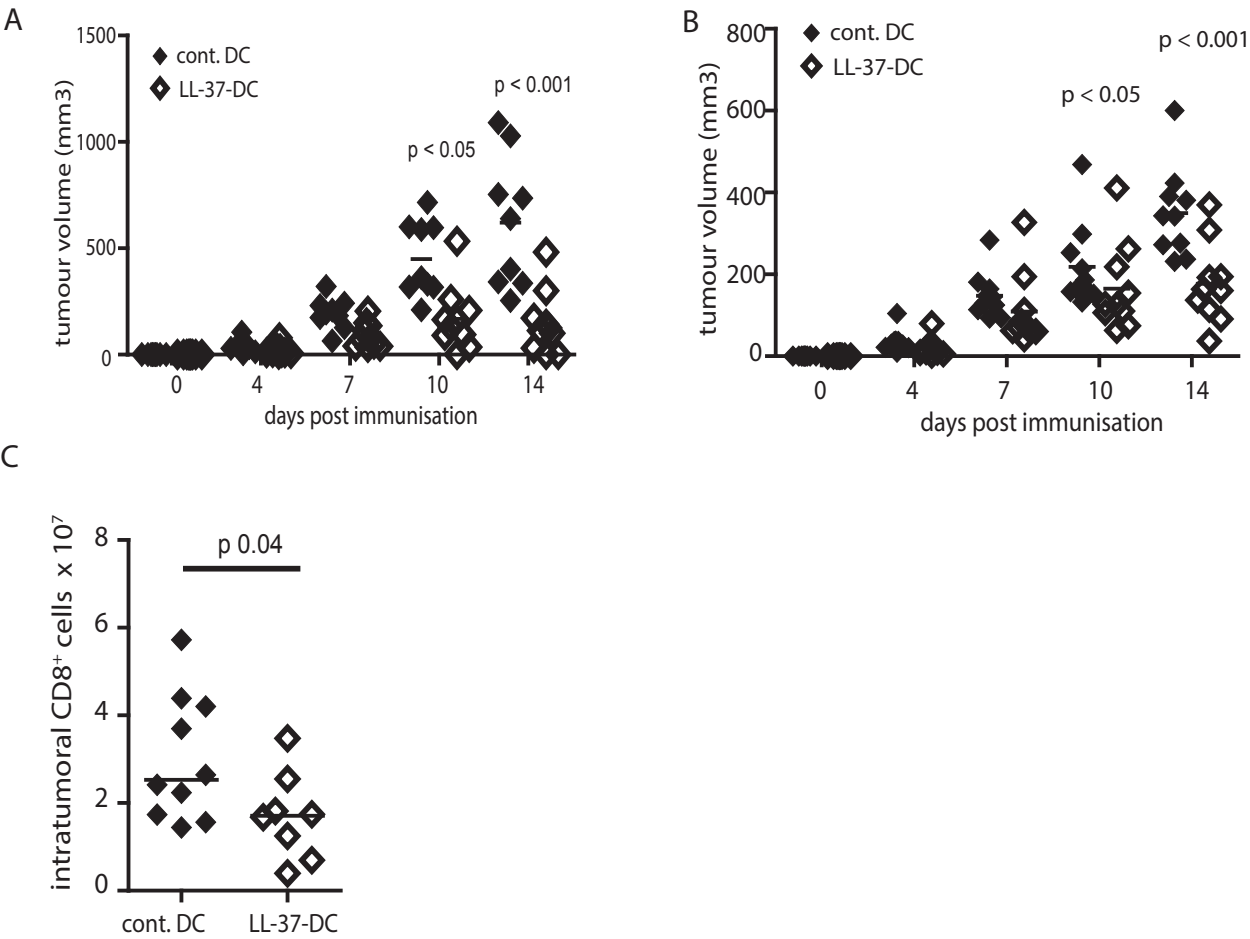

Supplement: Supplemental Material [file koni-08-08-1608106-s002.pdf]

Supplementary Figure 1

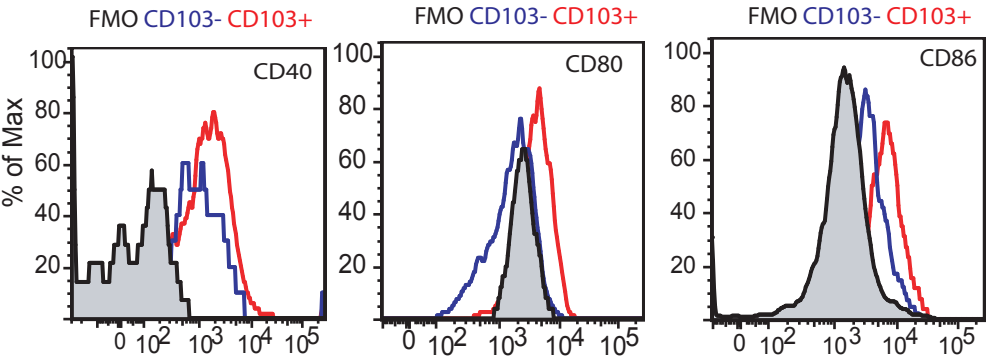

Supplement: Supplemental Material [file koni-08-08-1608106-s003.pdf]
